# A pilot trial to examine the association between circulating endothelial cell levels and vascular injury in patients with diabetes and chronic kidney disease

**DOI:** 10.12688/f1000research.8005.1

**Published:** 2016-03-07

**Authors:** Shayan Shirazian, Candace Grant, Vikash Rambhujun, Ritika Sharma, Ronak Patel, Shahidul Islam, Joseph Mattana

**Affiliations:** 1 Division of Nephrology, Department of Medicine, Winthrop University Hospital, Mineola, NY, USA; 2 Winthrop Research Institute, Winthrop University Hospital, Mineola, NY, USA

**Keywords:** Circulating Endothelial Cells, Diabetes, Chronic Kidney Disease, Vascular Injury

## Abstract

**Objective**

While albuminuria is a marker for progressive chronic kidney disease (CKD) in patients with type 2 diabetes (T2DM), both albuminuric and normoalbuminuric patients appear prone to vascular injury. This pilot study examines the association between circulating endothelial cell (CEC) levels and vascular injury in patients with T2DM and CKD.

**Methods**

In this cross-sectional study, eligible adult patients had T2DM, and stage 3 CKD (estimated glomerular filtration rate between 30 and 60 mL/min/1.73m ^2^). CEC levels were tested by Janssen Diagnostics, LLC using an immuno-magnetic bead-based assay. CEC levels were compared to levels in a previously tested normal population. Correlations between CEC levels and other vascular injury markers (urine albumin, von-Willebrand factor antigen, hs-CRP, uric acid) were performed.

**Results**

Patients included 40 adults of which nineteen were normoalbuminuric.  Mean CEC levels (38.7, SD 38.1 cells) were significantly higher than the normal population (M = 21±18 cells, p<0.001; N = 249), including in the normoalbuminuric subgroup (M = 42.9±42.5 cells, p<0.001). CEC levels were significantly correlated with uric acid levels (r=0.33, p=0.039).

**Conclusions**

CEC levels in patients with T2DM and CKD, both albuminuric and normoalbuminuric, are significantly higher than a normal population, suggesting the presence of vascular injury in both groups. Future studies are needed to evaluate the role of CECs as a biomarker to predict outcomes in normoalbuminuric patients with CKD.

## Introduction

Type 2 diabetes (T2DM) is the most common cause of pre-dialysis chronic kidney disease (CKD) in the United States affecting approximately 10 million Americans ^1, 2^. Of these patients with T2DM and CKD only a small proportion will progress to end stage renal disease (ESRD) requiring dialysis or transplant ^2^. Identifying patients with T2DM and CKD at high risk for progressive renal disease could lead to a decrease in the incidence of ESRD through the implementation of interventions known to slow progression ^3^. Unfortunately, identifying which of these patients are at high risk remains a challenge, due to inaccurate biomarkers of progressive renal disease.

Urine microalbumin levels are the most commonly used biomarker for progressive diabetic renal damage ^4^. Despite this, they have been found to be a poor predictor of progression to ESRD in T2DM, and are in the normal range in up to 50% of patients with T2DM and CKD ^5– 11^. Patients with diabetes and normoalbuminuria are at risk for progressive renal function decline ^12^, and those with diabetes, CKD and normoalbuminuria are at higher risk of cardiovascular events and all-cause mortality than diabetes patients without CKD ^13^. Furthermore, patients with T2DM, CKD and normoalbumuria have a similar prevalence of micro and macroangiopathies as patients with T2DM and albuminuria ^7^. Biomarkers that quantify underlying vascular injury and predict future renal and cardiovascular risk independent of urine microalbumin are needed in this population ^7, 14^.

Circulating endothelial cells (CECs) are a reliable serologic marker of vascular injury. These cells have been found to detach and circulate in human sera after sustained activation by inflammatory and thrombotic stimuli ^15^. Levels of these cells correlate with the degree of vascular injury, with higher levels seen in patients with atherosclerotic cardiovascular disease, small vessel vasculitis, renal transplant rejection, metastatic carcinoma and advanced chronic kidney disease ^15– 18^. In patients with T2DM and CKD, CEC levels may quantify the degree of underlying vascular damage independent of urine microalbumin, potentially acting as a seromarker for future renal or cardiovascular events. The purpose of this study was to determine whether CEC levels correlate with other markers of vascular injury in patients with T2DM and CKD including those with normoalbuminuria.

## Methods

### Study design and setting

This was designed as a cross-sectional study of CEC levels and other vascular injury markers in patients with T2DM and CKD. Recruitment occurred at the outpatient nephrology offices of a large northeastern hospital. This office has an annual caseload of approximately 1500 patients with T2DM and CKD, of whom 25% are minorities (black or hispanic). The study was approved by the local institutional review board (local board reference number 13002, IRBNet ID – 403557), and all patients gave their written informed consent prior to study procedures.

### Study population

Patients aged 18–89 years with T2DM for ≥ 8 years, and stage 3 CKD with an estimated GFR between 30 and 60 mL/min/1.73m ^2^ were considered for study inclusion. This inclusion criterion selected for a population at high risk for vascular injury that had not yet progressed to advanced CKD. Patients with prior evidence of renal artery stenosis, biopsy proven non-diabetic etiology of kidney disease, recurrent urinary tract infections, microhematuria, active malignancy, recent hospitalization and nephrotic range proteinuria were excluded. The exclusion criterion selected for patients with the highest likelihood of having diabetes related renal vascular injury, minimizing the possibility of elevations in CEC levels caused by alternative etiologies. Patients were selected to ensure approximately equal numbers of males and females, and normoalbuminuric and albuminuric subgroups.

Twelve hundred electronic charts with the ICD 9 coding diagnoses of T2DM and stage 3 CKD were selected for review of inclusion/exclusion criteria. Of these charts, 800 patients met all inclusion/exclusion criteria and 103 consecutive patients were asked to participate in the study when they presented for a follow-up appointment at our outpatients offices. Of those patients who were approached, 40 (38%) signed consent forms and participated in a single study session apart from their regularly scheduled follow-up visit.

### Study procedures

All study procedures were performed during a single, morning 1-hour study session. For all patients, a complete history and physical including seated blood pressure was performed and baseline characteristics were recorded. For blood pressure readings, the lower of two seated measurements taken after 5 minutes of sitting was recorded. Comorbidities were defined by patient report or documentation in the patient’s problem list. Cardiovascular disease was defined as a prior history of coronary artery disease (CAD), congestive heart failure (CHF), peripheral vascular disease (PVD) or stroke.

Blood and urine samples were collected from all patients at the time of recruitment. This included serum creatinine, uric acid, von Willebrand Factor antigen levels, high sensitivity C-reactive protein, morning urine microalbumin to creatinine ratios (UACR) and CEC levels. For low density lipoprotein (LDL) cholesterol, hemoglobin A1c (HbA1c), serum phosphorus and blood hematocrit, the most recent value from outpatient blood work taken within 6 months of the study visit was recorded. Patients with UACR < 30mg/g were classified as normoalbuminuric, while patients with a UACR ≥ 30mg/g were classified as albuminuric.

### Circulating endothelial cell measurement

Levels of total CECs were measured by the Clinical Lab Services Group at Janssen Diagnostics, LLC (previously Veridex Corporation) using the CELLSEARCH® Circulating Endothelial Cell Kit (Janssen Diagnostics, LLC). This system has provided valid and reproducible CEC levels in normal populations and in patients with chronic illness and malignancy, and the details have previously been described in detail ^19, 20^. Briefly, peripheral whole blood was obtained with a 21-G butterfly needle and the first 3mL was discarded to minimize falsely elevated CEC levels related to venipuncture ^19^. Whole blood was then collected into a tube containing anticoagulant and preservative, maintained at 15–30°C and shipped overnight to the central laboratory where it was processed within 72 hours. Prior studies have shown no significant difference in CEC levels at 0, 24, 48 and 72 hours in normal patients ^19^. The CEC levels in a normal healthy population from this study are quoted from previous studies performed using the CELLSEARCH® Circulating Endothelial Cell Kit (Janssen Diagnostics, LLC) ^19^.

CECs were isolated and enumerated by Janssen Diagnostics, LLC using the CELLSEARCH® Circulating Endothelial Cell Kit (Janssen Diagnostics, LLC) in combination with the CELLTRACKS® AUTOPREP® System (Janssen Diagnostics, LLC), and the CELLTRACKS ANALYZER II® (Janssen Diagnostics, LLC). These systems use immunomagnetic bead technology and a semi-automated fluorescent microscope. Briefly, 4mL of blood were mixed with 10mL of dilution buffer containing 0.1% sodium azide and the mixture was centrifuged at 800g for 10min, placed in the sample preparation system, i.e. the CELLTRACKS® AUTOPREP® System (Janssen Diagnostics, LLC), and processed within 1 hour of preparation. The sample preparation system then aspirated the plasma, leaving the buffy coat layer and red cells to be further processed. An anti-CD146 ferrofluid from a 3.0mL suspension of 0.012% magnetic nanoparticles conjugated to a mouse monoclonal antibody was added (CELLSEARCH® Circulating Endothelial Cell Kit; Janssen Diagnostics, LLC). The cells were then incubated inside the sample preparation system at room temperature for 12 to 13 minutes, at which time they underwent magnetic separation. The unbound cells and remaining plasma were then aspirated by the sample preparation system, and staining reagents with a permeablization buffer, to fluorescently label immunomagnetically bound cells, were added. These staining reagents included: 1) 4,6-diamidino-2-phenylindole (DAPI), a nuclear stain, 2) the mouse monoclonal CD105-PE which is specific for the protein endoglin and is expressed by endothelial cells; activated monocytes, stromal cells and pre-B cells, and 3) the mouse monoclonal pan-leukocyte antibody CD45 conjugated to allophycocyanin, in conjuction with a permeabilization buffer (CELLSEARCH® Circulating Endothelial Cell Kit; Janssen Diagnostics, LLC). Cells were then re-incubated and magnetic separation was repeated. Finally, the cells were re-suspended in approximately 300 µL of buffer and transferred to a chamber with two magnets that magnetically oriented cells into a monolayer for analysis. Analysis was performed within 24 hours using the CELLTRACKS ANALYZER II (Janssen Diagnostics, LLC), a four-color semi-automated fluorescent microscope and a gray scale charged coupled device camera, capturing 175 frames per channel. The captured frames were analyzed by image analysis software. CECs candidates selected by the software were then manually identified by the operator as DAPI+, CD105+ and CD45- cells. A single CEC count was performed for each blood specimen.

### Statistical analysis

The aim of this pilot trial was to study the feasibility of recruitment, and assessment procedures. The sample size of 40 was based on the feasibility of single center recruitment and the study budget. All normally distributed continuous variables are reported as mean±standard deviation, and all non-normally distributed variables as median (1 ^st^ quartile – 3 ^rd^ quartile). Categorical variables are reported as proportions. Kolmogorov-Smirnov test was used to evaluate the normality of all continuous variables. A dichotomous grouping variable was created using urine microalbumin variable (‘albuminuric’ if urine microalbumin >30 mg/g, ‘normo-albuminuric’ if <=30 mg/g). Two independent samples t-test was used to compare normally distributed variables between albuminuric and normo-albuminuric groups and Wilcoxon rank-sum test was used for non-normally distributed variables. Fisher’s exact test was used to compare all categorical variables. Correlations analyses between levels of CECs and other vascular injury markers were performed using Pearson’s correlation for normally distributed continuous variables and Spearman’s for non-normally distributed continuous variables. There were three missing data points in this study (von Willebrand factor antigen × 1, low density lipoprotein X 2). This missing data was ignored from analysis. All analyses were performed using SAS 9.4. Results were considered significant if p<0.05.

## Results

### Baseline characteristics

The characteristics of our study population are listed in Table 1. The average age of our group was 69.7 years, 52% were female, 72% were white and 20% were black ( Table 1). A history of cardiovascular disease was present in 43% of participants, with a history of CAD in 35%, CHF in 8%, PVD in 10%, and stroke in 0%. Mean levels of all vascular injury markers were significantly higher than the reference range for our laboratory. Characteristics of albuminuric and normoalbuminuric subgroups are listed in Table 1. Only diabetes duration was significantly different between subgroups.

**Table 1.** Demographics and clinical characteristics.

Variable	Overall (n=40)	Normoalbuminuric (n=19)	Albuminuric (n=21)	p-value ^‡^
Age, years, mean±SD	69.7±8.8	70.7±9.2	68.7±8.5	0.486
Gender, n(%)				0.342
Male	19(48)	8(42)	13(62)	
Female	21(52)	11(58)	8(38)	
Race, n(%)				0.281
Black	8(20)	6(32)	2(10)	
White	29(72)	12(63)	17(81)	
Asian	3(8)	1(5)	2(10)	
BMI, kg/m _2_, mean±SD	32.2±5.6	33.5±5.5	31±5.5	0.167
DM Duration, years, median(Q1-Q3) ^†^	16(10-20)	11(9-19)	19(16-20)	0.007
CVD, n(%)	17(42.5)	7(36.8)	10(47.6)	0.539
SBP, mmHg, mean±SD	126.8±13.7	125.3±15	128±13	0.478
DBP, mmHg, mean±SD	69.5±9.4	67.5±9.55	72±9	0.154
eGFR, ml/min/1.73m ^2^, mean±SD	45.9±12.5	48.5±11.3	43.6±13.5	0.224
vWF antigen, %, median(Q1-Q3) ^†^	200(165-222)	197(162-218)	203(166-223)	0.632
hsCRP, mg/L, median(Q1-Q3) ^†^	3.9(1.1-8.6)	2.7(0.8-8.4)	4(2.0-8.8)	0.588
LDL, mg/dL, mean±SD	89.4±40.9	89.4±42.6	89.5±40.3	0.996
HbA1c, %, mean±SD	7.3±1.2	7.5±1.3	7.2±1.1	0.429
Hematocrit, %, mean±SD	37.0±5.3	37.2±4.4	36.9±6.2	0.877
Serum uric acid, mg/dL, mean±SD	7.1±1.6	6.6±1.8	7.6±1.4	0.063

Note. BMI, body mass index, DM, diabetes mellitus, CVD, cardiovascular disease, SBP, systolic blood pressure. DBP, diastolic blood pressure, eGFR, estimated glomerular filtration rate using the 4-variable modified diet in renal disease equation ^21^, vWF, vonWillebrand factor, hsCRP, high sensitivity C-reactive protein, LDL, low-density lipoprotein. HbA1c, hemoglobin A1c

^†^Variables are not normally distributed. Hence, comparisons were performed using Wilcoxon rank-sum test.

^‡^p-values are from independent samples t-test for normally distributed continuous variables, Wilcoxon rank sum test for non-normally distributed variables and Fisher’s Exact test for categorical variables

### Circulating endothelial cell levels in normoalbuminuric and albuminuric groups

**Figure 1.**
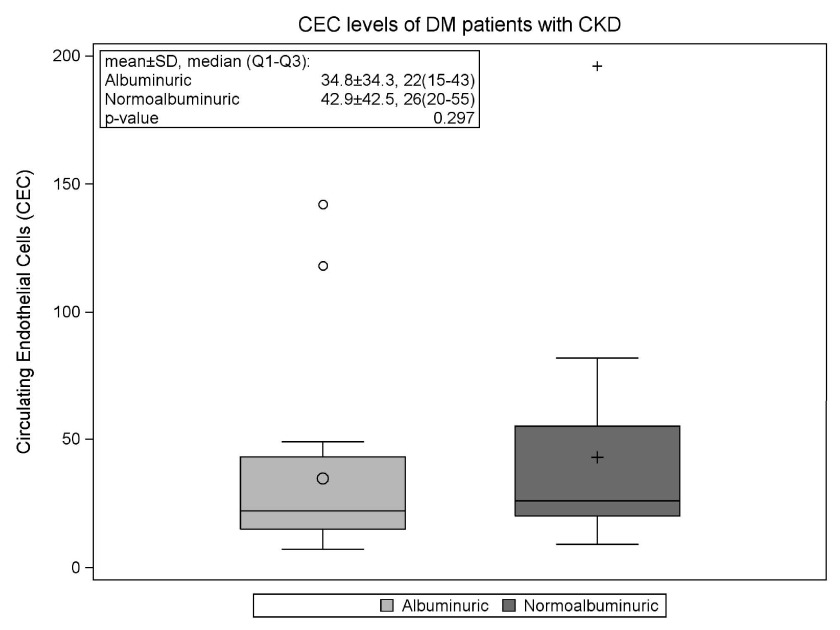
CEC levels of T2DM patients with CKD by albuminuria status. Box plots shows 25th, 50th (median) and 75th percentiles (horizontal bars). The lower fence is 1.5 × inter-quartile range (IQR) below the 25th percentile. The upper fence is 1.5 × IQR above the 75th percentile. The circle and plus signs inside the boxes are means. The circles outside the fences are outliers. Abbreviations: CEC, circulating endothelial cells; T2DM, type 2 diabetes mellitus; CKD, chronic kidney disease.

The mean CEC level in our study patients was 38.7±38.1 cells, range 7–196 ( Figure 1). These levels are significantly higher than levels tested in a historical normal population. In that study of 249 healthy subjects, done by Immunicon Corporation using the CELLSEARCH System®, the mean CEC level was 21±18 cells, range 0–97 ^19^. In our albuminuric subgroup, the average CEC level was 34.8±34.3 and in the normoalbuminuric subgroup the average CEC level was 42.9±42.5 ( Figure 1). There was no significant difference in CEC levels between the albuminuric and normoalbuminuric groups (p=0.297).

### Correlation between circulating endothelial cell levels and other vascular injury markers

Table 2 lists the correlation between CEC levels and other vascular injury markers tested in our study. We found a significant correlation between CEC levels and uric acid levels (r=0.33, p=0.039). No other significant correlations were found.

**Table 2.** Correlation of demographics and clinical variables with CEC levels.

Variable	r	p-value ^‡^
Age (years)	-0.13	0.409
SBP, mmHg	0.15	0.359
DBP, mmHg	0.05	0.752
BMI (kg/m ^2^)	0.17	0.298
DM Duration (years) ^†^	-0.03	0.833
Urine microalbumin, mg/g	-0.15	0.347
eGFR, mL/min/1.73m ^2^	0.13	0.413
vWF antigen, % ^†^	0.19	0.244
hsCRP, mg/L ^†^	0.02	0.929
LDL, mg/dL	0.03	0.845
HbA1c, %	0.20	0.216
Hematocrit, %	0.12	0.479
Serum uric acid, mg/dL	0.33	0.039

Note. CEC, circulation endothelial cells, SBP, systolic blood pressure. DBP, diastolic blood pressure, BMI, body mass index, DM, diabetes mellitus, eGFR, estimated glomerular filtration rate using the 4-variable modified diet in renal disease equation (21), vWF, vonWillebrand factor, hsCRP, high sensitivity C-reactive protein. LDL, low-density lipoprotein. HbA1c, hemoglobin A1c. Hct, hematocrit

^†^Non-normally distributed variables

^‡^p-values are from Pearson correlation coefficient for normally distributed variables and Spearman correlation coefficient analyses for non-normally distributed variables

## Discussion

In this study, we measured levels of CECs in patients with T2DM and CKD. We found that CEC levels are significantly higher than a normal population, and that there was no difference in these levels between normoalbuminuric and albuminuric subgroups. Additionally, CEC levels were significantly correlated with uric acid levels.

CEC levels have been found to be elevated in patients with chronic kidney disease and patients that have received a kidney transplant. In a study of CEC levels in 29 patients undergoing hemodialysis, 10 patients with diabetes and stage 1 or 2 CKD, 7 patients with hypertension and stage 1 or 2 CKD and 22 healthy patients, Koc *et al.* found that CEC levels were significantly higher in the group on dialysis and in the groups with diabetes and hypertension compared to the healthy controls. Furthermore, these levels were significantly higher in dialysis patients with active compared to stable atherosclerotic cardiovascular disease ^18^. We similarly found that CEC levels are higher in patients with T2DM and CKD when compared to CEC levels in a historical normal population. Additionally, our study is the first to demonstrate that CEC levels are elevated in normoalbuminuric patients with T2DM and CKD.

Our finding of elevated CEC levels in normoalbuminuric patients suggests severe vascular injury despite normoalbuminuria. This finding has been seen in prior studies of patients with diabetes and CKD. Cross-sectional studies have shown no significant differences in the frequencies of diabetic retinopathy, coronary heart disease cerebrovascular disease or peripheral vascular disease between patients with normo, micro or macroalbuminuria and CKD ^7, 14^. Furthermore, renal biopsy studies of patients with T2DM, CKD and normoalbuminuria have revealed a higher prevalence of vascular injury compared to biopsies of patients with micro and macroalbuminuria ^22– 24^.

Our results may have important clinical implications. We found a correlation between CEC levels and uric acid, a validated marker of vascular injury that has been linked to all-cause and cardiovascular mortality in patients with CKD ^25^. This finding suggests that CEC levels may also provide information about future vascular risk in patients with T2DM and CKD, and we believe warrant future studies examining this association. Recent prospective studies have validated cerebral and renal ischemia, as measured by cerebral MRI and renal ultrasound, as biomarkers of future renal risk in patients with T2DM and CKD independent of urine microalbumin ^26, 27^. If larger studies confirm our findings, then a future prospective study testing the ability of CEC levels to predict renal and cardiovascular outcomes, even in normoalbuminuric CKD patients, may be warranted.

Our pilot study is limited by its size and its cross-sectional design. Our goal for this study was to establish feasibility of patient recruitment and study procedures. Any observed associations need to be replicated in a larger-scale study, and none of the observed associations can be considered causal. Additionally, due to the small sample size, our results may not be applicable to the general CKD population.

In conclusion, we found that CEC levels are elevated in patients T2DM and CKD independent of urine microalbumin. These levels correlate with uric acid, a known marker of vascular injury. Larger, prospective studies are needed to confirm these findings.

## Data availability

The data referenced by this article are under copyright with the following copyright statement: Copyright: © 2016 Shirazian S et al.

*Harvard Dataverse*: Dataset. Shirazian, Shayan, 2016, “Replication Data for: A pilot trial to characterize circulating endothelial cells in patients with type 2 diabetes and chronic kidney disease”, http://dx.doi.org/10.7910/DVN/I64SAC ^28^

## Consent

Written informed consent for publication of their clinical details was obtained from all participants.
