# Supplementary material for: A pilot trial to examine the association between circulating endothelial cell levels and vascular injury in patients with diabetes and chronic kidney disease
Source: F1000Res. 2016 Mar 7;5:292. [Version 1] doi: 10.12688/f1000research.8005.1 (PMC4892336; doi:10.12688/f1000research.8005.1)
Supplement: Peer review history

# Peer review: Referee response for version 1

**DOI:** 10.5256/f1000research.8614.r14122

**Published:** 2016-06-02

**Authors:** Bernard Canaud

**Affiliations:** 1 Fresenius Medical Care, Bad Homburg, Germany

This pilot study was performed in 40 patients type 2 diabetes mellitus (T2DM)  and stage 2 chronic kidney disease (CKD) and examine the association between circulating endothelial cell (CEC) levels and  surrogates biomarkers of vascular damage (urine albumin, von-Willebrand factor antigen, hs-CRP, uric acid). Mean CEC levels were significantly higher (x1.8) than the normal population independently for albuminuria level and correlated with uric acid levels. Higher CEC levels have been already reported in severe renal disease (T1 and T2DM, CKD, Lupus…) and cardiovascular disease (ischemic cardiac disease, angioplasty coronary stenting or bypassing). CEC are considered as markers of severe vascular damage with poor outcome.

   

 The originality of this study is to identify that CEC levels are elevated also in normoalbuminuric patients with T2DM and CKD suggesting severe vascular injury despite the absence of significant albuminuria.

 Unfortunately, the predictive value of CEC levels either on cardiovascular disease or chronic kidney disease progression is missing. In addition, it would have been interesting to have data on endothelial progenitor cell (EPC) levels, which are markers of endothelial regeneration with vascular protective role. 

 As the authors mentioned, further prospective studies are needed to define the role and the predictive value of CEC in T2DM and CKD patients as biomarker of cardiovascular and kidney disease progression.

I have read this submission. I believe that I have an appropriate level of expertise to confirm that it is of an acceptable scientific standard.

# Peer review: Referee response for version 1

**DOI:** 10.5256/f1000research.8614.r13202

**Published:** 2016-04-06

**Authors:** Albert Dreisbach

**Affiliations:** 1 Department of Medicine, University of Mississippi Health Care, Jackson, MS, USA

Novel pilot study demonstrating significant increase in circulating vascular endothelial cells (CEC) in stage 3 chronic kidney disease with and without microalbuminuria suggesting significant vascular disease in patients with normal albuminuria and albuminuria compared to historical healthy controls. Significant correlation was also shown between CEC and serum uric acid.

This is a preliminary study and prospective outcome studies will need to be performed to demonstrate any clinical predictive power of CECs. This limitation the investigators acknowledge.

It would be interesting to measure CEC levels in T2DM without CKD (normal eGFR without microalbuminuria) and follow them prospectively to see whether they are predictive of development of CKD.

I have read this submission. I believe that I have an appropriate level of expertise to confirm that it is of an acceptable scientific standard.
